# A salt-regulated peptide derived from the CAP superfamily protein negatively regulates salt-stress tolerance in *Arabidopsis*

**DOI:** 10.1093/jxb/erv263

**Published:** 2015-06-20

**Authors:** Pei-Shan Chien, Hong Gil Nam, Yet-Ran Chen

**Affiliations:** ^1^Molecular and Biological Agricultural Sciences Program, Taiwan International Graduate Program, Academia Sinica, Taipei 115, Taiwan; ^2^Agricultural Biotechnology Research Center, Academia Sinica, Taipei 115, Taiwan; ^3^Graduate Institute of Biotechnology, National Chung-Hsing University, Taichung 402, Taiwan; ^4^Center for Plant Aging Research, Institute for Basic Science, and Department of New Biology, DGIST, Daegu 711-873, Republic of Korea

**Keywords:** Environmental regulation, CAP, negative regulator of salt resistance, plant peptide, proteolytic process, salinity.

## Abstract

High salinity has negative impacts on plant growth through altered water uptake and ion-specific toxicities. Plants have therefore evolved an intricate regulatory network in which plant hormones play significant roles in modulating physiological responses to salinity. However, current understanding of the plant peptides involved in this regulatory network remains limited. Here, we identified a salt-regulated peptide in *Arabidopsis*. The peptide was 11 aa and was derived from the C terminus of a *c*ysteine-rich secretory proteins, *a*ntigen 5, and *p*athogenesis-related 1 proteins (CAP) superfamily. This peptide was found by searching homologues in *Arabidopsis* using the precursor of a tomato CAP-derived peptide (CAPE) that was initially identified as an immune signal. In searching for a CAPE involved in salt responses, we screened CAPE precursor genes that showed salt-responsive expression and found that the *PROAtCAPE1* (*AT4G33730*) gene was regulated by salinity. We confirmed the endogenous *Arabidopsis* CAP-derived peptide 1 (AtCAPE1) by mass spectrometry and found that a key amino acid residue in PROAtCAPE1 is critical for AtCAPE1 production. Moreover, although *PROAtCAPE1* was expressed mainly in the roots, AtCAPE1 was discovered to be upregulated systemically upon salt treatment. The salt-induced AtCAPE1 negatively regulated salt tolerance by suppressing several salt-tolerance genes functioning in the production of osmolytes, detoxification, stomatal closure control, and cell membrane protection. This discovery demonstrates that AtCAPE1, a homologue of tomato immune regulator CAPE1, plays an important role in the regulation of salt stress responses. Our discovery thus suggests that the peptide may function in a trade-off between pathogen defence and salt tolerance.

## Highlight

An 11 aa peptide derived from one of the *c*ysteine-rich secretory proteins, *a*ntigen 5, and *p*athogenesis-related 1 (CAP) superfamily is salt regulated, conferring salt susceptibility through suppression of salt-tolerance genes.

## Introduction

Excessive salinity has a highly negative impact on plant growth due to the increased osmotic potential of soil and the toxicity of salt ions to cells (Bernstein, 1975; Munns and Tester, 2008). Accordingly, plants have developed various mechanisms for growth and survival in harsh saline environments. Extensive research has revealed an array of salinity regulatory mechanisms at the molecular level (Kreps *et al.*, 2002; Taji *et al.*, 2004), including changes in a vast number of genes encoding ion channels, transporters, transcription factors, signalling molecules, detoxification enzymes, chaperones, and osmolytes (Wang *et al.*, 2003; Munns and Tester, 2008; Turan *et al.*, 2012), that can be utilized to induce salt tolerance in crop plants (Turan *et al.*, 2012; Roy *et al.*, 2014). A systems-level study also revealed that the salt response involves an intricate web of regulatory networks that crosstalk with other stress (Wang *et al.*, 2003; Mahajan and Tuteja, 2005) and developmental (Bernstein, 1975) responses. Among the regulation signals for high salt, phytohormones are thought to be the most important endogenous substances for modulating physiological responses that eventually lead to adaptation to salinity (Pearce *et al.*, 2001b). Recently, plant peptides have been found to function as hormones in a diverse array of roles in plant growth, development, and signalling to environmental stresses (Matsubayashi, 2014). However, to date, little has been reported on plant peptides that regulate the salt response (Matsubayashi, 2014).

Recent research based on studies of precursor genes has suggested a functional link between plant peptides and modulation of the salt-stress response. A knockout mutant of one of the C-TERMINALLY ENCODED PEPTIDE (CEP) family proteins exhibited more tolerance to salinity than wild-type *Arabidopsis*, and external application of the peptide to seedlings suppressed root growth (Ohyama *et al.*, 2008; Delay *et al.*, 2013). This suggests the relevance of plant peptides functioning in root development in the regulation of underground stresses such as salinity. Additional evidence showed that tomato with overexpression of PROSYSTEMIN, the precursor of the anti-herbivore systemin peptide signal (Pearce *et al.*, 1991), displayed more tolerance to salt stress than wild-type tomato (Pearce *et al.*, 1991; Orsini *et al.*, 2010). This finding suggests that defence-related peptides may also participate in the modulation of salt-stress tolerance.

We recently identified a peptide derived from tomato pathogenesis-related protein 1 (PR1) that is wound induced in the same way as systemin and regulates immune responses (Chen *et al.*, 2014). Since PR1 protein was classified as one of the *c*ysteine-rich secretory proteins, *a*ntigen 5, and *p*athogenesis-related 1 proteins (CAP) superfamily (Gibbs *et al.*, 2008), the peptide derived from the protein in this superfamily was designated CAP-derived peptide (CAPE) (Chen *et al.*, 2014). Notably, CAPE was found to be wide spread throughout the plant kingdom, being found in dicots (e.g. *Solanoideae*, *Nicotianoideae*, *Vitaceae*, *Brassicaceae*, and *Fabaceae*) and monocots (e.g. *Poaceae*), with a conserved ·PxGNxxxxxPY- motif (Chen *et al.*, 2014). It has been demonstrated that application of a synthetic peptide predicted to be a CAPE derived from *Arabidopsis* PR1 can lead to resistance against *Pseudomonas syringae* pv. *tomato* strain DC3000 (Pst DC3000) infection (Chen *et al.*, 2014). This finding indicates that *Arabidopsis* CAP proteins may produce functional CAPEs. Moreover, overexpression of *Arabidopsis* PR1 and tomato PROSYSTEMIN showed drought and salt-stress tolerance, respectively (Seo *et al.*, 2008; Wang *et al.*, 2011; Liu *et al.*, 2013), which led us to test the hypothesis that *Arabidopsis* CAPEs may function not only in regulation of innate immunity but also in the response to salt-stress tolerance. In this report, we aimed to identify the endogenous *Arabidopsis* CAPEs that are involved in salt responses and we showed that a CAPE negatively regulates the salt-tolerance response by downregulating salt-tolerance genes upon high-saline challenge. The phenotypic evidence and the CAPE-mediated salt-responsive mechanism were studied and are discussed.

## Materials and methods

Details of the seedling germination assay, RNA extraction and quantitative reverse transcription PCR (qRT-PCR) analysis, and western blotting are provided in the Supplementary materials and methods, available at *JXB* online.

### Plant materials and growth conditions

A T-DNA mutant of AT4G33730 (GT_5_110620; named *proatcape1* here) was obtained from the Nottingham *Arabidopsis* Stock Centre (NASC) for phenotypic investigation. *Arabidopsis* ecotype Landsberg erecta (Ler) was used in all experiments as the background control for *proatcape1* and PROATCAPE1ox in *proatcape1* transgenic lines generated in a *proatcape1* background. In addition, *Arabidopsis* ecotype Columbia-0 (Col-0) was used as the background control for CAPE1ox^CNYD^ and CAPE1ox^CNAD^ transgenic plants. A schematic representation of the target site of *PROAtCAPE1* by T-DNA is shown in Supplementary Fig. S2A (available at *JXB* online). Homozygous *proatcape1* was determined by genotyping (Supplementary Fig. S2B). qRT-PCR results indicated that *proatcape1* was a knockout mutant (Supplementary Fig. S2C). The primers used for PCR-based genotyping are listed in Supplementary Table S2, available at *JXB* online.

Seeds were surface sterilized with 30% bleach (CLOROX) for 8min and then washed with sterilized ddH_2_O five times. Seeds were germinated on half-strength Murashige and Skoog (1/2 MS) medium under a 16h photoperiod (80–100 μmol m^−2^ s^−1^ illumination) at 22 °C.

### Plasmid constructions and generation of transgenic lines

Total RNA extracted from Ler was utilized as the template for reverse transcription to generate cDNA. The cDNA was used as template for PCR using primers (Supplementary Table S2) corresponding to the 5′ and 3′ ends of the *PROAtCAPE1* coding sequence. The amplified DNA fragment was cloned into pCR8/GW/TOPO (Invitrogen) according to the manufacturer’s protocol. The validated *PROAtCAPE1* sequence was then cloned into pMDC32 (Curtis and Grossniklaus, 2003), a constitutive expression vector harbouring a dual 35S promoter, by site-specific recombination (LR clonase; Invitrogen). Similarly, for the generation of CAPE1ox^CNYD^ transgenic plants, the *PROAtCAPE1* sequence was recombined into pK7YWG2 (Karimi *et al.*, 2002). For the generation of p*PROAtCAPE1*:GUS lines, the sequence upstream of the transcription start site of *PROAtCAPE1* (2kb) was cloned into pCR8/GW/TOPO and then recombined into pMDC164 (Curtis and Grossniklaus, 2003). Transgenic *Arabidopsis* was generated via an *Agrobacterium tumefaciens*-mediated transformation system (Zhang *et al.*, 2006).

### Microarray analysis

Quality control of total RNA was determined using an Agilent Bioanalyzer 2100 (Agilent Technologies, St Clara, CA, USA). Total RNA (0.2 μg) was amplified by a Low Input Quick-Amp Labeling kit (Agilent Technologies). Preparation of fluorescence-labelled cDNA and microarray experiments were performed at the DNA Microarray Core Facility, Institute of Plant and Microbial Biology, Academia Sinica, Taiwan. Agilent *Arabidopsis* (V4) Gene Expression Microarray 4×44k chips were used in this study. Labelling of cDNA probes and hybridization experiments were performed according to the single-colour microarray protocols provided by the manufacturer. The fluorescence signals were detected by an Agilent DNA Microarray Scanner G2565CA and Agilent Feature Extraction 10.7.1.1 software. Three biological repeats were conducted using cDNAs obtained from Ler and *proatcape1* in 1/2 MS medium and with 12h of 125mM NaCl treatment. Raw data from hybridization were imported into microarray analysis software GeneSpring 11.5 (Agilent Technologies), and the data of Ler subjected to medium alone was used as a control for normalization. As a quality control, we kept the genes whose raw signal intensity was >100 and coefficient of variation (CV) was <50% (default value) at any time point for further analyses. The coefficient of variation is a statistical measurement to compare the degree of variation from one data set to another, even if the means differ dramatically from each other.

The microarray data discussed in this publication have been deposited in the Gene Expression Omnibus of the National Center for Biotechnology Information (NCBI) (Edgar *et al.*, 2002) and are accessible through GEO Series accession number GSE66946.

### Endogenous peptide isolation

Ten-day-old Ler seedlings grown vertically on 1/2 MS medium were transferred to a hydroponic system culture of 1/2 MS liquid medium without sucrose. The medium was changed every 4 d. Three weeks after planting, the plant tissue was harvested. For salt treatments, fresh 1/2 MS medium in the presence or absence of 125mM NaCl was exchanged and the treatments were prolonged to 24h. Samples were homogenized with 200ml of 1% (v/v) chilled trifluoroacetic acid (TFA; Sigma-Aldrich) in a blender for 2min. To normalize the variations in sample preparation and for better quantification accuracy by liquid chromatography–tandem mass spectrometry (LC-MS/MS) analysis, 20 μl of 1 pmole μl^–1^ of internal standard (synthetic peptide: PAAAYIGARAY) with two amino acid substitutions of G3A and N4A of AtCAPE1 was added to the extraction buffer while crude peptide was isolated. The extracts were then filtered through four layers of Miracloth (Calbiochem, San Diego, CA, USA) to remove plant debris. The procedure followed a previously described protocol (Pearce *et al*., 2001*a*, *b*). After centrifuging at 8500rpm for 20min at 4 °C (Beckman Coulter, Avanti J-26 XP), the pH value of the supernatant was adjusted to 4.5. The extracts were then centrifuged again at 8500rpm for 20min at 4 °C and the pH value of supernatant was adjusted to 2.5. Subsequently, the supernatant was bound with a customized Sep-Pak C18 solid phase extraction cartridge (Waters, Milford, MA, USA) according to the following steps: the C18 cartridge was conditioned by 0.1% (v/v) TFA first, bound with the supernatant, and then washed with 0.1% (v/v) TFA. Finally, the polypeptides were eluted with 60% (v/v) methanol/0.1% (v/v) TFA. The peptides in the final eluate were evaporated to dryness by rotary evaporation under high vacuum, and the pellets were resuspended in 0.1% (v/v) TFA. The polypeptides were further fractionated by fast protein liquid chromatography (ÄKTA purifier) to remove protein contaminates with size exclusion columns (Superdex peptide 10/300 GL; GE Healthcare, Little Chalfont, UK). Fractions containing polypeptides or small proteins with similar sizes to AtCAPE1 were collected and combined. The combined eluates were rotary evaporated under high vacuum and resuspended in 0.1% (v/v) TFA for ZipTip (Millipore, Billerica, MA, USA) to remove the contamination of salts. After final evaporation and resuspension in 0.1% (v/v) formic acid (Fluka), targeted LC-MS/MS analysis was performed.

### Targeted LC-MS/MS analysis

An LTQ Velos PRO mass spectrometer (Thermo Scientific, Waltham, MA, USA) coupled with an online capillary nanoUHPLC system (Waters) was utilized for peptide identification and quantification. The capillary LC system was equipped with a homemade C18 trap cartridge (5 μm particles, Symmetry C18; Waters), and a homemade C18 reversed-phase analytical column (1.7 μm particles, BEH130 C18; Waters) (Chen *et al.*, 2012) was used to deliver the solvent and target peptide with a linear gradient from 8 to 90% (v/v) acetonitrile in 0.1% (v/v) formic acid for 95min at a nanoflow rate (approx. 300 nl min^–1^). The analytical column was coupled to a nanoelectrospray ionization source, and acquisition of the data was performed with a full MS scan followed by MS/MS scans of the targeted precursor ions. Precursor ions of AtCAPE1 (PAGNYIGARPY; *m*/*z* 589.8) and the internal standard (PAAAYIGARAY; *m*/*z* 562.4) were selected for subsequent targeted MS/MS scans. The fragment ions *m*/*z* 563.2, 676.3, and 900.5 and *m*/*z* 537.2, 650.3, and 813.34 were used for further identification and quantification of AtCAPE1 and the internal standard, respectively.

### Relative quantification of endogenous AtCAPE1

Quantitative analysis of AtCAPE1 was carried out using a label-free approach. Values of the peak area extracted from the selected fragment ions in the extracted ion current chromatogram were calculated. The sum of three peak values represented the expression of the target peptide. After normalizing AtCAPE1 to the internal standard in each sample, the relative area ratio for AtCAPE1 displayed a comparison of AtCAPE1 expression in different samples with that in shoots under normal conditions.

## Results

### Identification of PROAtCAPE as specifically responding to salt stress in *Arabidopsis*

Putative CAP proteins in *Arabidopsis* were searched using the precursor of tomato CAP-derived peptide (CAPE1), the first peptide derived from the CAP protein superfamily in plants (Chen *et al.*, 2014), as a query protein for a homology search against the TAIR10 protein database. Twenty-two *Arabidopsis* CAPs were discovered that displayed sequence similarity (E-value <1) with the tomato CAPE1 precursor. To further refine the candidate proteins that may produce CAPEs, the 22 potential *Arabidopsis* CAP proteins were searched by the C-terminal conserved motif, CNYx.PxGNxxxxxPY- (Chen *et al.*, 2014) (Table 1). Nine potential CAPs were identified as precursor candidates for CAPEs. These proteins were named PROAtCAPEs and their putative CAPEs, containing the ·PxGNxxxxxPY- motif, were named AtCAPEs (Table 1). The nomenclature followed the order of percentage identity between the putative AtCAPEs and tomato CAPE1 (Chen *et al.*, 2014). Thus, the putative AtCAPE derived from AT4G33730 was designated AtCAPE1 and its precursor as PROAtCAPE1. The putative AtCAPEs derived from the known pathogenesis-related proteins PRB1 and PR1 were named AtCAPE7 and AtCAPE9, respectively (Table 1).

**Table 1.** List of putative CAPEs in Arabidopsis

Peptide name^a^	Putative CAPE sequence^b^	Gene symbol^c^
AtCAPE1	.PAGNYIGARPY-	AT4G33730
AtCAPE2	.PPGNWVGEWPY-	AT4G25780
AtCAPE3	.PPGNWVGEWPY-	AT4G33720
AtCAPE4	.PPGNYVGEKPY-	AT4G25790
AtCAPE5	.PPGNYVGEKPY-	AT5G57625
AtCAPE6	.PPGNFLGRKPY-	AT4G30320
AtCAPE7	.PPGNYANQKPY-	PRB1
AtCAPE8	.PPGNYRGRWPY-	AT5G26130
AtCAPE9	.PRGNYVNEKPY-	PR1

^*a*^ Peptide name given in the present work.

^*b*^ Putative CAPEs were defined by the conserved sequence. ·PxGNxxxxxPY- at the C terminus of the CAP proteins.

^*c*^ The gene symbol refers to NCBI nomenclature.

To search potential salt-responsive CAPEs, the expression of the nine *PROAtCAPE* genes that responded to salt stress were examined by public microarray data analysis (Hruz *et al.*, 2008). A probe for *PROAtCAPE5* was not available in the Affymetrix GeneChip (Supplementary Table S1, available at *JXB* online). Excluding *PROAtCAPE5*, the eight *PROAtCAPE* transcripts were identified as responding to salt and salt-related stresses according to our criteria of expression fold change of ≥1.5 (up- or downregulated) (*P*≤0.05; Supplementary Table S1). In the roots, *PROAtCAPE1* transcripts were decreased upon salinity, the *PROAtCAPE3* gene was decreased upon salinity, osmotic, drought, and cold stresses, the *PROAtCAPE4* gene was downregulated by salt and osmotic stresses, and the *PROAtCAPE6* gene was suppressed by salt, osmotic, and drought stresses, while *PROAtCAPE7* (also known as PRB1) was upregulated upon oxidative and cold stresses (Supplementary Table S1). In the shoots, *PROAtCAPE1* transcripts were increased upon salinity, the *PROAtCAPE2* gene was decreased upon saline and drought stresses but increased under cold stress, the *PROAtCAPE3* gene was upregulated by salt; *PROAtCAPE6* transcripts were suppressed by oxidative stress, and *PROAtCAPE9* (also known as PR1) was downregulated upon salinity, oxidative, drought and cold stresses (Supplementary Table S1). Notably, *PROAtCAPE1* was regulated mainly by salt, while the transcripts of the other seven *PROAtCAPE*s were regulated by more than two abiotic stresses (Supplementary Table S1). This analysis implied that AtCAPE1 derived from PROAtCAPE1 may be more specific to the salt-stress response than the other eight AtCAPEs. We therefore focused our further investigation on the role of the potential AtCAPE1 in salt-stress responses.

### AtCAPE1 is endogenous in *Arabidopsis*

**Fig. 1.**
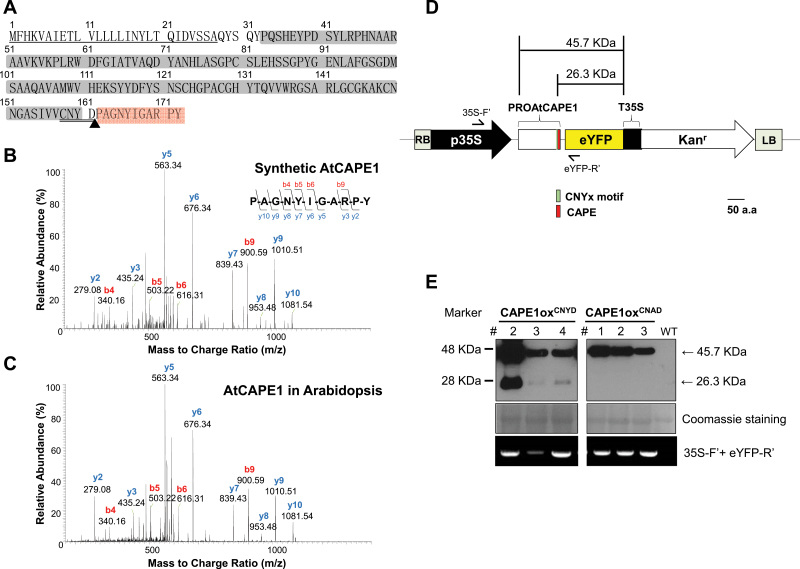
Identification of AtCAPE1 in *Arabidopsis*. (A) Deduced amino acid sequence of the PROAtCAPE1 product. The predicted secretion signal peptide is underlined. The CAP domain is shaded in grey. The putative AtCAPE1 peptide is shaded in red. The cleavage site predicted to produce AtCAPE1 is indicated with an arrowhead. The putative cleavage signal motif is double-underlined. (B) LC-MS/MS spectrum of the synthetic AtCAPE1. The y-ion is the C-terminal fragments after peptide bond cleavage while the b-ion is the N-terminal fragments. (C) LC-MS/MS spectrum of the identified AtCAPE1 in *Arabidopsis*. (D) Schema representing the construct used for constitutive overexpression of enhanced yellow fluorescent protein (eYFP)-tagged PROAtCAPE1. The green box shows the CNYx motif. The putative CAPE is shown in red. The numbers indicate the predicted molecular weight of precursor protein tagged with eYFP (45.7kDa) and the cleaved precursor tagged with eYFP (26.3kDa). (E) Production of the precursor PROAtCAPE1 and the cleaved PROAtCAPE1 in CAPE1ox^CNYD^ and CAPE1ox^CNAD^ transgenic plants, where eYFP was fused to PROAtCAPE1 containing wild type (CNYD) and the mutated (CNAD) junction sequence, respectively. T3 seedlings derived from independent transgenic lines were sampled for western blotting with anti-GFP antibody. Coomassie blue staining was used for protein loading control. The lower panel shows the presence of the T-DNA insertions in the transgenic plants by genomic DNA PCR with the primer pair 35S-F’ and eYFP-R’ shown in (D).

The deduced coding region of the PROAtCAPE1 product was 172 aa (Fig. 1A). A predicted signal peptide was found at the N terminus with a cleavage site between aa 27 and 28 (SignalP 4.1 Server, http://www.cbs.dtu.dk/services/SignalP/), suggesting that PROAtCAPE1 is a secretory protein that is synthesized in the endoplasmic reticulum (ER) and secreted into the extracellular space. The CAP domain (Gibbs *et al.*, 2008) conserved among CAPs from various species was located in the middle of the sequence (aa 43–160), while the putative AtCAPE1 was at the C-terminal end (Fig. 1A).

To find endogenous evidence of AtCAPE1 in *Arabidopsis*, we performed nanoflow LC-MS/MS (nanoLC-MS/MS) operated in MS/MS full scan mode (Chen *et al.*, 2012). After optimizing the collision-induced dissociation energy using synthetic AtCAPE1 as the standard, the condition attained a detection limit of ~10 attomole in our system. For the synthetic standard, 80% y-ion and 40% b-ion series coverage for the peptide fragments were observed in the MS/MS spectrum (Fig. 1B). In the analysis of endogenous peptides extracted from wild type (Ler), one MS/MS spectrum showed all the b- and y-ions matched to the standard. The ID confidence for a full-scan MS/MS spectrum is above the criteria for ID assignment that was proposed by the European Commission and noted in the ‘COMMISSION DECISION of 12 August 2002 implementing Council Directive 96/23/EC concerning the performance of analytical methods and the interpretation of results’. Therefore, the result indicated the existence of endogenous AtCAPE1 (Fig. 1C).

Among the PROAtCAPE proteins, the short conserved CNYx motif juxtaposing the CAPE sequence was assumed to be the cleavage signal that generates the CAPEs (Fig. 1A) (Chen *et al.*, 2014). To confirm this proteolytic process of the AtCAPE1 precursor, we generated a transgenic plant, CAPE1ox^CNYD^, where wild-type PROAtCAPE1 fused with C-terminal enhanced yellow fluorescent protein (eYFP) was constitutively overproduced by the 35S promoter (Fig. 1D). Expression of PROAtCAPE1 was examined in three independent CAPE1ox^CNYD^ transgenic lines by probing with anti-green fluorescent protein (GFP) antibody (Fig. 1E). In all of the three transgenic lines, we observed the presence of two bands with a molecular weight (MW) close to 48 and 28kDa, indicated by the protein marker (Fig. 1E, left-hand panel). The MWs of the two bands were close to that of the precursor protein tagged with eYFP (45.76kDa) and the putative AtCAPE1 fused to eYFP (26.3kDa), respectively (Fig. 1E, left-hand panel), when the cleavage occurred at the predicted cleavage site (Fig. 1A). We then generated transgenic lines, named CAPE1ox^CNAD^, where eYFP was fused with PROAtCAPE1 but with a mutation (Y160A, Fig. 1A) in the conserved cleavage motif. We then examined expression of the mutated PROAtCAPE1–eYFP. Only a single band with a MW of 45.76kDa was detected in all three independent transgenic lines (Fig. 1E, right-hand panel). Taken together, these results suggested that the identified AtCAPE1 was derived from its precursor, PROAtCAPE1, through cleavage at the conserved CNYx motif, and that an aromatic amino residue, tyrosine, is important for the process.

### Expression of the *PROAtCAPE1* gene is downregulated by salt stress

**Fig. 2.**
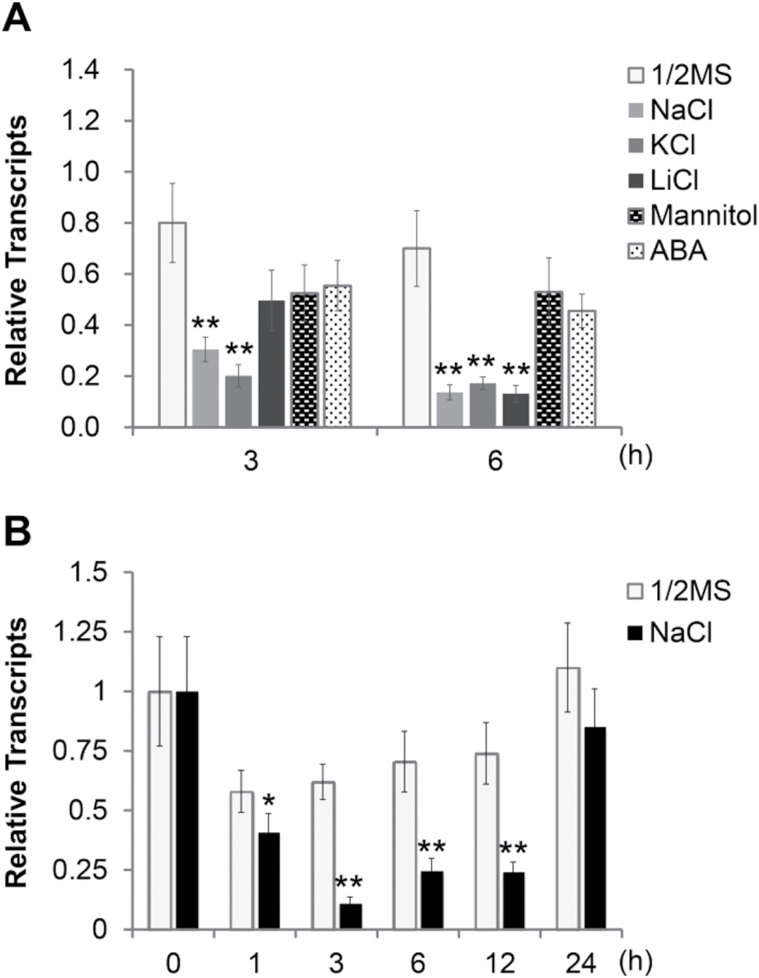
Levels of *PROAtCAPE1* transcripts under salinity. (A) Ten-day-old wild-type (Ler) seedlings were subjected to 150mM NaCl, 150mM KCl, 30mM LiCl, 300mM mannitol, and 100 μM abscisic acid (ABA). Relative transcripts indicate the normalized *PROAtCAPE1* level (*PROAtCAPE1*/*ACTIN2*) from each sample compared with that in wild type in medium alone (1/2MS) at 0h. The bar shown here is the mean of three biological repeats. Error bars indicate means±SEM (stress-treated wild-type versus untreated wild-type at different time points; Student’s *t*-test: **P≤0.01, *P≤0.05). (B) Ten-day-old wild-type seedlings were treated without (1/2MS) or with 125mM NaCl. Zero hours means that the seedlings were subjected to medium alone and harvested immediately. The bars shown here are the means from four biological repeats. Error bars indicate means±SEM (salt-treated wild-type versus untreated wild-type at different time points; Student’s *t*-test: **P,≤0.01, *P,≤0.05).

Having confirmed the endogenous presence of AtCAPE1, we further validated that expression of the precursor gene, *PROAtCAPE1*, was regulated by salt stress via qRT-PCR (Fig. 2A). In comparison with normal growth condition, the transcripts of *PROAtCAPE1* were significantly reduced by around 3- to 4- fold (*P*≤0.01) after 3 and 6h treatment with 150mM NaCl and KCl, as well as 6h treatment with 30mM LiCl (Fig. 2A). However, incubation with 300mM mannitol or 100 μM abscisic acid (ABA) for 3 and 6h showed no significant effect on the expression of the *PROAtCAPE1* gene (Fig. 2A). As expression of the *PROAtCAPE1* gene was significantly reduced upon incubation with three different salts, NaCl, KCl, and LiCl, but with not mannitol, we propose that the reduced expression of *PROAtCAPE1* under high salinity is mainly due to ionic stress, not osmotic stress. In addition, ABA showed much less effect on expression of *PROAtCAPE1* in comparison with the effect derived from salt ions, and the reduction in expression of the *PROAtCAPE1* gene was still observed in the ABA biosynthesis mutants (Supplementary Fig. S1, available at *JXB* online) *nced3*, *aba2*, and *aba3* (Nambara and Marion-Poll, 2005). These findings indicated that the *PROAtCPAE1* gene may not be regulated by an ABA-dependent pathway under salinity. A comparison of the expression level at 3 and 6h of incubation with 150mM NaCl revealed that the expression of *PROAtCAPE1* showed a kinetic response to salt stress. Therefore, more detailed kinetics of the expression of *PROAtCAPE1* were examined upon incubation with a slightly reduced salt concentration (125mM NaCl) (Fig. 2B). We found that the transcript level of *PROAtCAPE1* started to show a noticeable decrease in the first 1h, reaching a minimum at 3h (Fig. 2B). This result showed that the downregulation of *PROAtCAPE1* is an early cellular response to salt stress. Interestingly, the expression level remained low up to 12h but recovered to a level close to that of wild type at 24h (Fig. 2B; see Discussion).

### AtCAPE1 negatively regulates salt tolerance

To test the physiological role of AtCAPE1 in response to salt stress, the growth of wild-type (Ler) and *proatcape1* mutant (T-DNA inserted knockout line with Ler background; Supplementary Fig. S2) in normal growth medium and medium containing NaCl for 20 d was compared (Supplementary Fig. S3, available at *JXB* online). The *proatcape1* mutant seedlings did not show a noticeable defect under normal growth conditions. In 100mM NaCl, more surviving seedlings with green and large-sized leaves were observed in *proatcape1* than in the wild type (Supplementary Fig. S3). In 125mM NaCl, neither wild-type nor *proatcape1* seedlings survived. We also tested whether exogenously supplied synthetic AtCAPE1 could restore the salt response of the *proatcape1* mutant (Supplementary Fig. S3). The presence of 1 μM synthetic AtCAPE1 partially restored the salt response, as shown by the reduced growth in 100mM NaCl to a level close to that in wild type (Supplementary Fig. S3). These results indicated that AtCAPE1 confers salt sensitivity on *Arabidopsis* and thus acts negatively on the salt-tolerance response.

**Fig. 3.**
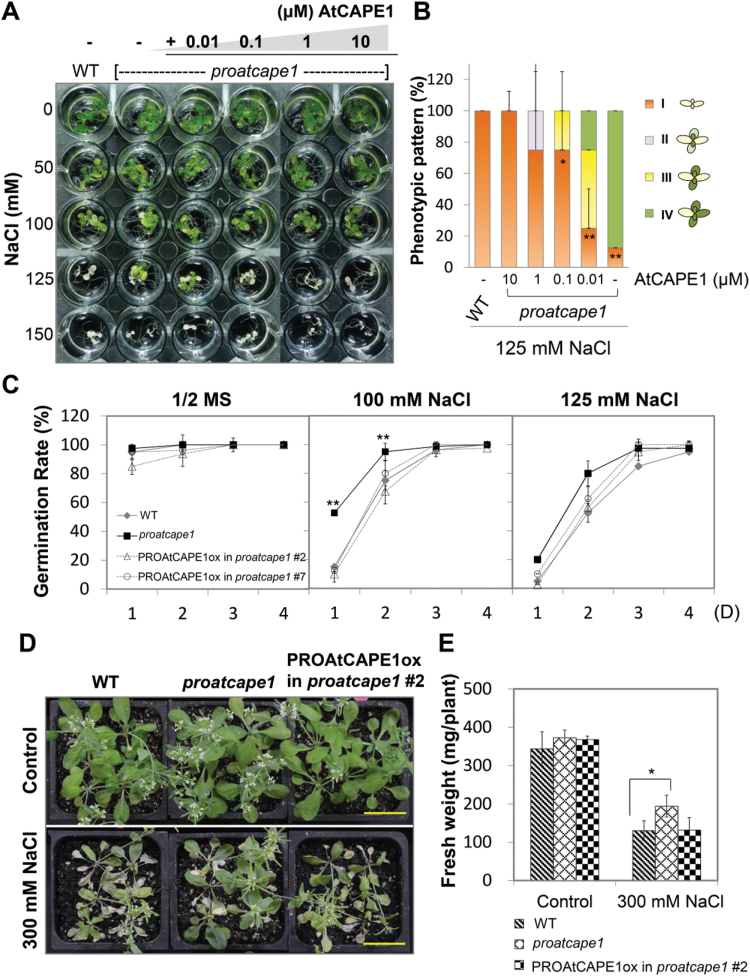
AtCAPE1 negatively regulates the salt-tolerance response. (A) Growth response of *proatcape1* mutants to salt stress and to AtCAPE1. Five-day-old seedlings were treated with various concentrations of NaCl in the absence (–) and presence (+) of various concentrations of synthetic AtCAPE1. The photograph was taken after treatment for another 10 d. (B) Semi-quantitative analysis of the phenotype of plants shown in (A). Each bar represents the mean percentage of the phenotypic pattern from two independent experiments. Error bars indicate means±SD. The phenotypic pattern according to leaf number and colour of seedlings was defined into categories I–IV as shown in the diagram (salt- and peptide- treated *proatcape1* versus salt-treated wild-type Ler; Student’s *t*-test: **P≤0.01, *P≤0.05). (C) Germination rates of *proatcape1* mutant and PROAtCAPE1ox in *proatcape1* transgenic plant seeds compared with the corresponding wild-type (Ler) seeds. Each value represents the percentage of germination (with 40 seeds) for four independent tests. Error bars indicate the means±SEM. (Student’s *t*-test: **P≤0.01, *P≤0.05). (D) Three-week-old plants were irrigated without (Control) and with 300mM NaCl three times for every second day. After that, plants were recovered with water for another one week. The photograph was taken then. (E) Shoot fresh weight was measured after treatments from (D). Data are shown as an average fresh weight from 36 plants. Error bars indicate means±SD. An asterisk indicates significant differences (*P≤0.05) between *proatcape1* mutant and wild-type Ler plants upon 300mM NaCl treatment.

Salt stress retards plant development and impairs seed germination (Greenway and Munns, 1980). We analysed these two responses in more detail in the *proatcape1* mutant. The growth response of the mutants was measured by subjecting 5-d-old wild-type and *proatcape1* seedlings to various concentrations of NaCl for a further 10 d (Fig. 3A). Although the treatment with 50 and 100mM NaCl caused the leaves of the wild-type and *proatcape1* seedlings to turn light green, no significant difference was observed between the two (Fig. 3A). Upon treatment with 125mM NaCl, *proatcape1* seedlings displayed greenish cotyledons and generated true leaves, whereas wild-type seedlings exhibited yellowish cotyledons and stunted growth (Fig. 3A). Neither Ler nor *proatcape1* seedlings survived following 150mM NaCl treatment (Fig. 3A). We further investigated the phenotype of salt-treated Ler and *proatcape1* subjected to various concentrations of peptides. Introduction of various concentrations (0, 0.01, 0.1, 1, and 10 μM) of synthetic AtCAPE1 into the medium did not cause any visible growth retardation. The effect of the peptide became clearly noticeable upon treatment with 125mM NaCl. Under this condition, a semi-quantitative analysis of the phenotypic severity observed with various concentrations of peptide was conducted by categorizing the plants into four classes. As shown in Fig. 3B, upon 125mM NaCl treatment, none of the wild-type seedlings survived (class I) after treatment for 10 d; similarly, the leaves of all the *proatcape1* seedlings turned totally yellow and stopped growing (class I) when subjected to an additional 10 μM synthetic AtCAPE1. In the presence of 1 and 0.1 μM peptide, 75% of the *proatcape1* seedlings died (class I) and 25% displayed either four yellow to pale green (class II) or four green (class III) leaves out of a total of six leaves, respectively (Fig. 3B). The peptide had a visible effect at concentrations as low as 0.01 μM (25% of seedlings in class I); when no peptide was supplemented, nearly 90% of the *proatcape1* mutants displayed five out of six green leaves (class IV) (Fig. 3B). The quantitative phenotype indicated that the negative effect caused by AtCAPE1 is dosage dependent.

We next examined the germination rates of wild-type and *proatcape1* mutant seeds on medium containing various concentrations of NaCl (Fig. 3C). When grown on a medium supplemented with 100mM NaCl, *proatcape1* seeds displayed significantly higher germination rates than wild-type (Ler) seeds at early time points (1–2 d, Fig. 3C). No significant difference was found when both types of seeds were sown on medium containing 125mM NaCl (Fig. 3C). To complement the phenotype of *proatcape1* under salinity, we generated a transgenic line of *proatcape1* harbouring a constitutive overexpression cassette for PROAtCAPE1 (named PROATCAPE1ox in *proatcape1*; Supplementary Fig. S2D). Two PROATCAPE1ox in *proatcape1* transgenic lines displayed a slower germination rate than *proatcape1* mutant seeds in the presence of 100mM NaCl for 1–2 d. Moreover, adult PROATCAPE1ox in *proatcape1* transgenic lines also displayed reduced resistance to salinity as compared with *proatcape1* mutants, with less fresh weight of shoots (Fig. 3D, E). These results indicated that overexpression of PROAtCAPE1 could counteract the salt resistance of the *proatcape1* mutant.

Taken together, the knockout mutant of *PROAtCAPE1* showed a reduced response in growth retardation and induced germination under high-salt conditions, whereas exogenous application of synthetic AtCAPE1 (Fig. 3A, B; Supplementary Fig. S3) or overexpression of peptide precursor (Fig. 3C–E) restored the salt response of *proatcape1* mutants. These results demonstrated that AtCAPE1 functions as a negative regulator of salt-stress tolerance in *Arabidopsis*.

### Transcriptome analysis reveals that AtCAPE1 negatively regulates the transcripts of salt-inducible genes

**Fig. 4.**
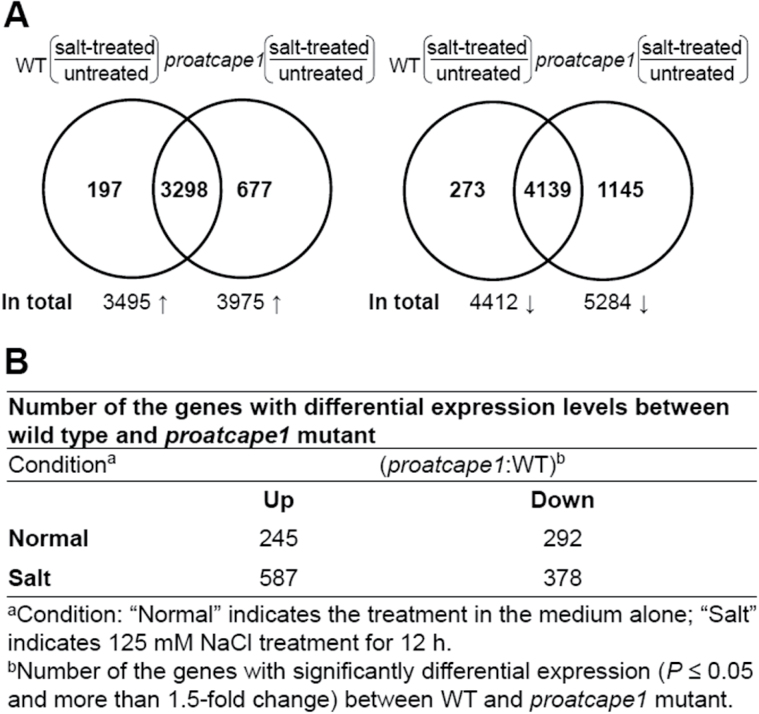
Number of genes differentially expressed in wild type (Ler) and the *proatcape1* mutant under high salt. (A) Numbers indicate the genes with significantly differential expression (*P*≤0.05 and a greater than 1.5-fold change) between the indicated data sets derived from microarray analysis. (B) Number of genes with differential expression levels between wild type and the *proatcape1* mutant under normal and salt (12h of treatment in 125mM NaCl) conditions (*P*≤0.05 and a greater than 1.5-fold change).

To gain insight into the mechanism by which AtCAPE1 regulates salt responses, we investigated the gene expression profiles of wild-type (Ler) and *proatcape1* mutant seedlings in the presence and absence of 125mM NaCl by microarray analysis (Fig. 4). First, we identified 3495 and 4412 genes that were up- and downregulated, respectively, by over 1.5-fold in the wild type after 12h of treatment in 125mM NaCl (Fig. 4A; Supplementary dataset I, available at *JXB* online). This observation was in agreement with previous reports that salt treatment leads to a large change in the gene expression profile and is involved in many physiological processes (Kreps *et al.*, 2002; Seki *et al.*, 2002; Jiang and Deyholos, 2006). In the mutant, 3975 and 5284 genes were up- and downregulated, respectively, by over 1.5-fold under salinity (Fig. 4A; Supplementary dataset II, available at *JXB* online). The result showed that the overall sensitivity of plants to salt stress was increased in the mutant, as more genes were affected in the mutant than in the wild type. We then compared the genes that were differentially expressed in the wild type and mutant. Under normal conditions, 245 and 292 genes were up- and downregulated by over 1.5-fold in the mutants (Fig. 4B; Supplementary dataset III, available at *JXB* online). In contrast, after 12h of treatment with 125mM NaCl, 587 and 378 genes were up- and downregulated by over 1.5-fold in the mutants (Fig. 4B; Supplementary dataset IV, available at *JXB* online). The result showed that more genes were upregulated under high salinity. As the mutant displayed salt tolerance, it is likely that these upregulated genes in the mutant under high salinity contribute to the salt-tolerant phenotype. As analysed by Gene Ontology (GO) (Huang *et al*., 2009*a*, *b*), the gene products of these genes could be described by GO terms including ‘response to water deprivation’, ‘response to abscisic acid stimulus’, and ‘response to salt stress’ (Table 2).

**Table 2.** GO analysis ^*a*^ of the 587 differentially upregulated genes in proatcape1 under salt conditions

Biological function^*b*^	Number of genes^*c*^	*P* value
Cellular amino acid derivative metabolic process	21	2.51E–06
Phenylpropanoid metabolic process	20	2.61E–09
Lipid biosynthetic process	19	3.61E–03
Cellular lipid metabolic process	19	1.48E–02
Cellular amino acid derivative biosynthetic process	18	9.05E–07
Aromatic compound biosynthetic process	18	3.73E–06
Phenylpropanoid biosynthetic process	17	1.36E–08
Response to salt stress	16	1.32E–02
Response to fungus	16	4.95E–02
Response to abscisic acid stimulus	15	3.32E–03
Response to water deprivation	13	4.05E–04
Response to reactive oxygen species	12	2.08E–04
Response to bacterium	12	1.60E–02
Innate immune response	12	3.47E–02
Response to cold	11	2.85E–02

^*a*^ The selected salt-responsive genes were uploaded into DAVID Bioinformatics Resources 6.7 (Huang *et al*., 2009*a*, *b* ) for GO analysis. The biological functions categorized by GO analysis were selected by level four.

^*b*^ The top 15 most abundant biological process categories are shown.

^*c*^ Number of genes (from a total of 587 genes) displayed for each category.

**Fig. 5.**
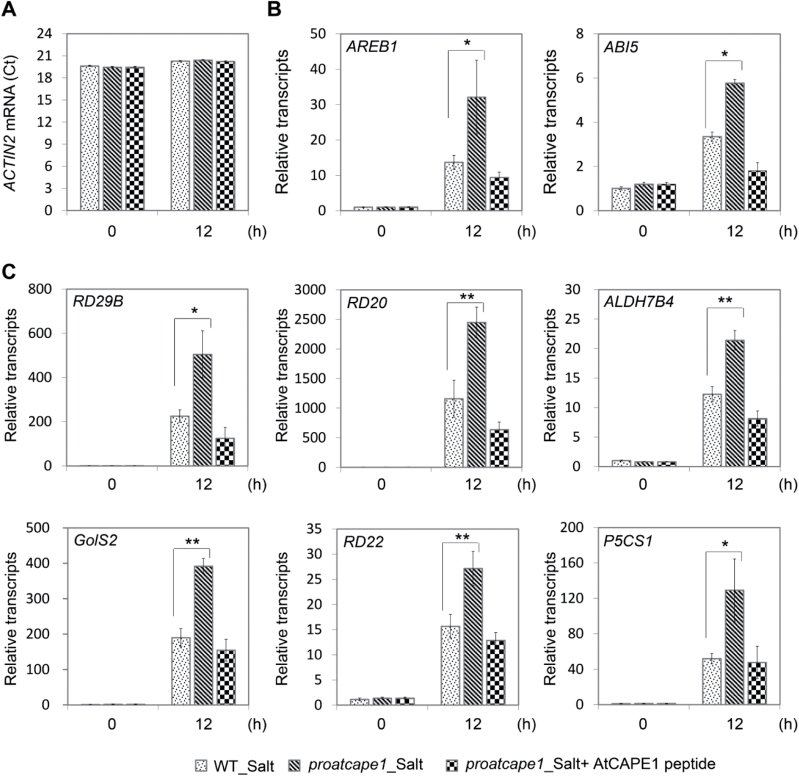
Expression of various salt-inducible genes is downregulated by AtCAPE1. Ten-day-old seedlings were treated without and with salt for 12h. The transcript levels of the selected salt-inducible genes in wild-type (Ler) and *proatcape1* mutant seedlings were determined by qRT-PCR: *ACTIN2* (A), *AREB1* and *ABI5* (B), and *RD29B*, *RD20*, *ALDH7B4*, *GolS2*, *RD22*, and *P5CS1* (C). Zero hours means that the seedlings were subjected to the medium indicated and harvested immediately. For *proatcape1*, the mutants were subjected to either 125mM NaCl (*proatcape1*_Salt) or 125mM NaCl in the presence of 500nM AtCAPE1 peptide (*proatcape1*_Salt+AtCAPE1 peptide). The mean values from four biological repeats are shown. Error bars are means±SEM (*proatcape1* versus Ler upon salt treatment; Student’s *t*-test: **P≤0.01, *P≤0.05).

We further confirmed some of the salt-inducible genes regulated by AtCAPE1. The *ACTIN2* transcript was used as an internal control in this experiment as its expression did not respond to salinity (Fig. 5A). *ABA-responsive element binding protein 1* (*AREB1*) (Fujita *et al.*, 2005) and *ABA-INSENSITIVE 5* (*ABI5*) (Finkelstein and Lynch, 2000) are well-characterized basic leucine zipper transcription factors involved in ABA signalling under drought and high-salinity conditions (Uno *et al.*, 2000; Nakashima and Yamaguchi-Shinozaki, 2013 m). The expression level of the *AREB1* gene was highly upregulated by salt by around 13-fold in the wild type (Fig. 5B), which is in agreement with previous reports (Uno *et al.*, 2000; Fujita *et al.*, 2005). The *proatcape1* mutation resulted in a further increase in *AREB1* of around 2-fold compared with that in the wild type under salinity. However, the increased expression of *AREB1* in the mutant was completely restored to the wild-type level by exogenous treatment with AtCAPE1 (Fig. 5B). A similar trend was found in the regulation of the *ABI5* gene (Fig. 5B), although the *ABI5* expression was low after seedling establishment (Nakashima and Yamaguchi-Shinozaki, 2013). AtCAPE1 also negatively regulated the expression of high-salt-inducible downstream genes, including the genes for the enzymes involved in osmoprotectant biosynthesis [*Δ* ^*1*^ *-PYRROLINE-5-CARBOXYLATE SYNTHASE 1* (*P5CS1*) (Yoshiba *et al.*, 1999); and *GALACTINOL SYNTHASE* (*GolS2*) (Taji *et al.*, 2002)], for detoxification [*ALDEHYDE DEHYDROGENASES 7B4* (*ALDH7B4*) (Kirch *et al.*, 2004; Kotchoni *et al.*, 2006)], and for the dehydration response [*RD22*, *RD20* (also known as *CLO3*), and *RD29B* (Yamaguchi-Shinozaki and Shinozaki, 1993; Ingram and Bartels, 1996; Takahashi *et al.*, 2000)] (Fig. 5C). Taken together, these results indicated that the negative role of AtCAPE1 in salt tolerance is through downregulation of the salt-tolerance genes involved in salt-stress resistance. A further investigation of the levels of *RD29B* transcripts regulated by various concentrations of AtCAPE1 (0.5, 5, 50, and 500nM and 5 μM) in *proatcape1* mutants upon salinity indicated that, when introducing 50–500nM AtCAPE1 to the mutants, the induced *RD29B* genes in mutant lines were suppressed to the same level as that of the wild type upon salt stress (Supplementary Fig. S4, available at *JXB* online). However, an increasing peptide concentration (5 μM) did not show more suppression (Supplementary Fig. S4). This result suggested that the AtCAPE1 level in wild-type plants in response to salinity may have reached the maximum suppression efficacy for *RD29B* expression.

### Production of AtCAPE1 is post-translationally regulated under saline conditions

**Fig. 6.**
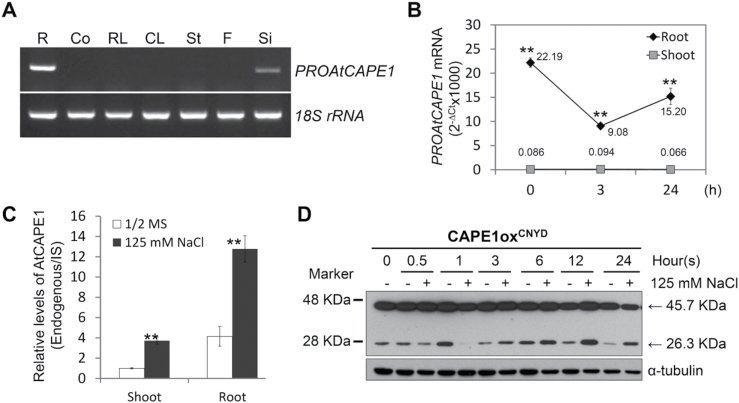
Production of AtCAPE1 is mainly derived from root tissues and is regulated by salt. (A) Transcriptional levels of *PROAtCAPE1* in different tissues were determined by RT-PCR. Total RNA was extracted from root (R), cotyledon (Co), rosette leaf (RL), cauline leaf (CL), stem (St), flower (F) and silique (Si). *18S rRNA* transcripts were used as internal control. (B) Salt response of the transcriptional levels of *PROAtCAPE1* in shoots and roots. Ten-d-old seedlings of wild type (Ler) were treated with 125mM NaCl for 0, 3, and 24h. The transcripts of *PROAtCAPE1* from the harvested roots and shoots were determined by RT-qPCR. Shown are the average values of (2^-∆Ct^x1000) from four biological repeats. Error bars, means±SE (*PROAtCAPE1* transcripts in shoots versus *PROAtCAPE1* transcripts in roots at different time points; Student’s *t*-test, **P≤0.01). (C) Relative level of endogenous AtCAPE1 in shoots and roots. Seedlings grown for 24h without (1/2 MS) and with 125mM NaCl were subjected to quantitative LC-MS/MS analysis. IS, internal standard. The average values from two biological repeats are shown. Error bars, means±SE. Asterisks indicate statistically significant differences between salt-treated and untreated samples (Student’s *t*-test; **P≤0.01). (D) Post-translational regulation of AtCAPE1 production. Protein extracts from the transgenic lines (CAPE1ox^CNYD^) harbouring the AtCAPE1-eYFP fusion grown with (+) and without (-) 125mM NaCl for the indicated times were subjected to western blot analysis. The upper and lower bands with approximate size of 45.7 KDa and 26.3 KDa represent the expected size of the PROAtCAPE1-eYFP fusion protein and the AtCAPE1-eYFP fusion protein, respectively. The fusion proteins were detected by anti-GFP antibody. α-tubulin, loading control.

A series of molecular mechanisms converting the signal into phenotypic resistance to salinity in plants is initiated in the root and systemically transmitted to the shoot (Munns and Tester, 2008; Roy *et al.*, 2014). Thus, the expression of *PROAtCAPE1* in different tissues was examined to reveal how *PROAtCAPE1* functions in response to salinity. Based on RT-PCR analysis, the *PROAtCAPE1* gene was found to be expressed most highly in the roots and was barely detected in above-ground tissues except for the silique (Fig. 6A). In comparison with the wild type under normal conditions, slightly shorter primary roots were observed in *proatcape1* seedlings (Supplementary Fig. S5E, available at *JXB* online). This phenotype suggested the possibility of a functional trade-off of AtCAPE1 between root growth and salt tolerance. When grown to the stage of inflorescence, no growth retardation and no abnormal morphology were observed in siliques in a comparison between the wild type and mutants (Supplementary Fig. S5, available at *JXB* online). A further investigation on roots by β-glucuronidase (GUS) staining showed that the promoter activity was observed in stelar cells from the elongation zone to the maturation zone and the trichoblast at the maturation zone (Supplementary Fig. S6, available at *JXB* online).

To elucidate whether *PROAtCAPE1* expressed in the roots was the main signal in response to salt stress, *PROAtCAPE1* transcripts were further examined separately in the shoots and roots of *Arabidopsis* seedlings in 125mM NaCl (Fig. 6B). In roots, the transcript level of *PROAtCAPE1* was downregulated within 3h, in agreement with our previous observation shown in Fig. 2. In shoots, the transcript was barely detected under the same conditions (Fig. 6B). GUS staining of 10-d-old p*PROAtCAPE1*:GUS transgenic lines in the absence and presence of salt for 24h reflected the mRNA expression pattern in the shoots and roots shown by qRT-PCR (Fig. 6B and Supplementary Fig. S7, available at *JXB* online). *PROAtCAPE1* was expressed mainly in the roots, yet mediated salt-tolerant phenotypic effects in the leaves (Fig. 3). We thus examined whether AtCAPE1 is produced in the roots and systemically transferred to shoots resulting in salt-stress responses throughout the whole seedling. Targeted LC-MS/MS analysis was performed (Chen *et al*., 2012, 2014) to quantify the level of endogenous AtCAPE1 in shoots and roots (Fig. 6C). We found that, under normal conditions, AtCAPE1 was present in the shoots at 25% of the level in the roots (Fig. 6C). Interestingly, in seedlings treated with salt for 24h, the level of AtCAPE1 was 4-fold and 3-fold higher in the shoots and in roots, respectively, than in the seedlings without salt treatment (Fig. 6C). Thus, the presence of AtCAPE1 in both tissues suggests that AtCAPE1 may function systemically.

As the transcription of *PROAtCAPE1* displayed the wild-type level after 24h of treatment in 125mM NaCl (Figs 2B and 6B), but AtCAPE1 was increased 3-fold (Fig. 6C), we tested whether the production of AtCAPE1 was regulated at the post-translational level. For this test, the transgenic plant CAPE1ox^CNYD^ (Fig. 1D, E) was utilized. In Fig. 6D, the putative AtCAPE1–eYFP (indicated at a MW of 26.3kDa) was reduced rapidly upon 125mM NaCl treatment, reaching a minimum at 1h, but then increased, being relatively higher than that without salt treatment. The western blot clearly demonstrated that the proteolytic process of PROAtCPE1 was dynamically regulated under saline conditions.

## Discussion

Here, we attempted to identify a salt-responsive peptide from *Arabidopsis*. We found a small peptide, AtCAPE1, by analysing the structure and expression patterns of the CAP superfamily proteins, that contained a CNYx.PxGNxxxxxPY- motif at the C terminus (Table 1). The predicted peptide existed endogenously and was 11 aa, making it the smallest currently known unmodified peptide signal in plants. The substitution of Y160A in the conserved CNYx motif completely blocked the processing of PROAtCAPE1 (Fig. 1E), demonstrating that the aromatic residue tyrosine is critical for the cleavage of PROAtCAPE1. A dibasic -RR- or -GR- motif is the only currently well-known protease recognition site located upstream of mature peptides in plants (Pearce and Ryan, 2003; Liu *et al.*, 2007; Matos *et al.*, 2008; Srivastava *et al.*, 2008). However, no -RR- or -GR- motif was observed preceding the CAPEs, suggesting that such motifs are not utilized for the cleavage of CAP proteins. This work, therefore, uncovered a unique protease recognition motif important for regulation of the proteolytic process. The signal peptide is predicted to be at the N-terminal PROAtCAPE1 protein sequence (Fig. 1A). Thus, PROAtCAPE1 may be secreted into the extracellular space and cleaved into mature AtCAPE1 in the apoplast (Delaunois *et al.*, 2013).

As the knockout mutant of *PROAtCAPE1* conferred salt tolerance to *Arabidopsis* seedlings, and exogenous application of AtCAPE1 to the mutant rendered the seedlings salt sensitive, this peptide is identified as a negative regulator of salt tolerance or a positive regulator of the salt-sensitive response in *Arabidopsis*. Based on our data showing the existence of the peptide (Fig. 6C, D), we envision that AtCAPE1, under normal conditions, suppresses the expression of salt-tolerance-related genes. This may be to avoid unnecessary expression of salt-tolerance genes in the absence of salt.

The level of the peptide was regulated dynamically upon salt treatment. Initially, following salt treatment, expression of *PROAtCAPE1* gene was rapidly downregulated and was recovered at later time points (Fig. 2B). Furthermore, processing of PROAtCAPE1 into mature peptides was also rapidly diminished (Fig. 6D). Together, these results showed that the reduction of AtCAPE1 level is an early response of plants to salt stress. This leads to temporary relief of expression of the salt-tolerance genes suppressed by AtCAPE1. However, after a prolonged period of salt treatment, expression of the *PROAtCAPE1* gene, proteolytic processing, and accordingly production of AtCAPE1 was increased to an even higher level than that in the wild type (Figs 2B, and 6C, D). The increased amount of the peptide then led to suppression of salt-tolerance genes and resulted in susceptibility to salt stress (Figs 3 and 5). However, in the *proatcape1* mutant with no AtCAPE1 production, the salt tolerance-related genes may be expressed constitutively, leading to better salt tolerance than in the wild type. Therefore, treatment of the mutant with exogenous AtCAPE1 suppressed expression of the salt-tolerance genes, recovering the salt sensitivity. Nonetheless, the expression level of AtCAPE1 may have reached maximum efficacy in terms of salt susceptibility in the wild type, because the salt-tolerance gene expression (Supplementary Fig. S4) and the salt susceptibility phenotype (Fig. 3A, B) in the mutant lines were only suppressed to the wild-type level, even when high concentrations (micromolar level) of AtCAPE1 were applied. In addition, overexpression of PROAtCAPE1 in transgenic lines only displayed a similar germination rate to the wild type under high salinity (Fig. 3C). This can be explained by the threshold effect of the peptide, and it is probable that protease activity for processing PROAtCAPE1 into mature AtCAPE1 is also a limiting effector.

Endogenous AtCAPE1 was present in the shoots at 25% of the level in the roots. AtCAPE1 detected in the shoots is less likely to be derived from its precursor since *PROAtCAPE1* gene expression was undetectable or extremely low in the shoots (Fig. 6B). Given the large discrepancy between the level of *PROAtCAPE1* transcripts in shoots and roots (Fig. 6A, B), AtCAPE1 detected in the shoots might have originated from the roots. The shoots of wild-type seedlings were more sensitive to salt than the mutant seedlings. In addition, the leaf-specific *RD20*/*CLO3* gene encoding a calcium-binding protein that signals the regulation of stomatal closure under stress (Aubert *et al.*, 2010) was regulated by AtCAPE1. We propose that, at least in part, the salt sensitivity of the wild-type shoot is mediated by the systemic peptide signal.

Our study further shed light on how AtCAPE1 mediates salt sensitivity or negatively regulates salt tolerance at the molecular level. As the microarray comparison between the wild type and the *proatcape1* mutant revealed, expression of hundreds of salt-responsive genes was altered in the mutants. A part of the molecular mechanism of AtCAPE1-mediated salt sensitivity can be explained as follows. The genes downregulated by AtCAPE1 included the transcription factors ABI5 and AREB1, which control the transcription of downstream ABA-dependent and salt-responsive genes. Both ABI5 and AREB1 are basic leucine zipper transcription factors; however, ABI5 functions in response to stresses mainly during germination, while AREB1 functions mainly at the vegetative stage (Nakashima and Yamaguchi-Shinozaki, 2013). Therefore, the delayed germination caused by AtCAPE1 may in part be governed by the ABI5-dependent signalling pathway, while the growth defects observed in seedlings are in part mediated by the AREB1-dependent regulon. AREB1 activates the downstream gene expression by binding to a conserved *cis*-acting ABA-responsive element (ABRE) in their promoter regions (Uno *et al.*, 2000). The salt-responsive genes *RD29B* and *RD20*/*CLO3* containing the ABRE element in the promoter region (Uno *et al.*, 2000; Fujita *et al.*, 2005; Aubert *et al.*, 2010) were found to be a direct target of AREB1 (Uno *et al.*, 2000; Fujita *et al.*, 2005). Since *P5CS1* and *ALDH7B4* also contain the *cis* ABRE (Yamaguchi-Shinozaki and Shinozaki, 1994; Yoshiba *et al.*, 1999; Missihoun *et al.*, 2014), it is highly likely that they are regulated by AREB1 as well. Thus, reduced expression of these downstream genes by AtCAPE1 may have been, in part, the result of reduced expression of AREB1 by AtCAPE1. The reduced expression of *P5CS1* may result in the reduced accumulation of proline, functioning as an osmolyte to balance water potential in plant cells (Ross *et al.*, 2004; Denoux *et al.*, 2008). Reduced expression of *ALDH7B4* would lead to a lesion in the detoxification processes of aldehydes that overaccumulate in plants when exposed to abiotic stresses (Kotchoni *et al.*, 2006) and to the susceptibility to NaCl treatments as observed in the *aldh7b4* mutant (Kotchoni *et al.*, 2006). We also found that AtCAPE1 downregulated the transcription of *GolS2*, which encodes a biosynthesis enzyme for the production of raffinose and galactinol, which function as osmoprotectants against high salinity (Taji *et al.*, 2002). Since there is no ABRE element in the promoter region of *GolS2* (Taji *et al.*, 2002), AtCAPE1-mediated downregulation of this gene is unlikely through AREB1.

We have unequivocally shown that AtCAPE1 negatively regulates salt tolerance or mediates salt sensitivity. Thus, what would be the possible advantage of having this endogenous peptide? From the phenotypic assays, we showed that *proatcape1* seedlings have slightly shorter primary roots than wild-type seedlings. This phenotype may indicate the possible involvement of the peptide in a trade-off between root growth and salt tolerance. We reported previously that a CAP-derived peptide could mediate an anti-pathogenic response in tomato (Chen *et al.*, 2014). It is thus also feasible that AtCAPE1 may be involved in a possible trade-off between pathogenic defence and salt tolerance. In addition, the knowledge that an endogenous peptide is a negative regulator of salt stress and information about the regulation of the proteolysis of a precursor protein into peptides can be utilized in strategies to introduce salt stress tolerance in plants by blocking the cleavage of precursor proteins.

## Supplementary data

Supplementary data are available at *JXB* online.

Supplementary materials and methods.

Supplementary Fig. S1. Levels of PROAtCAPE1 transcripts in the mutants of ABA biosynthesis genes under salinity.

Supplementary Fig. S2. Transcripts of *PROAtCAPE1* in transgenic plants.

Supplementary Fig. S3. AtCAPE1 negatively regulates salt tolerance response.

Supplementary Fig. S4. Application of AtCAPE1 peptide reduces the transcript levels of RD29B under salinity.

Supplementary Fig. S5. Phenotypic investigation of wild-type Ler and *proatcape1* mutant.

Supplementary Fig. S6. Expressions of PROAtCAPE1 in roots.

Supplementary Fig. S7. GUS-staining assay revealed the *PROAtCAPE1* promoter activity in the shoots and roots upon salt stress.

Supplementary Table S1. Effect of selected abiotic stresses on expressions of PROAtCAPEs.

Supplementary Table S2. Primers used in this study.

Supplementary dataset I. Differentially expressed genes in wild type upon the treatment in the presence and absence of 125mM NaCl for 12h.

Supplementary dataset II. Differentially expressed genes in *proatcape1* mutants upon the treatment in the presence and absence of 125mM.

Supplementary dataset III. Differentially expressed genes between *proatcape1* mutants and wild type in the normal condition.

Supplementary dataset IV. Differentially expressed genes between *proatcape1* mutants and wild type in the presence of 125mM NaCl for 12h.

Supplementary Data

## Abbreviations:

ABA: abscisic acid
Col-0: Columbia-0
ER: endoplasmic reticulum
eYFP: enhanced yellow fluorescent protein
GFP: green fluorescent protein
GO: Gene Ontology
GUS: β-glucuronidase
LC-MS/MS: liquid chromatography–tandem mass spectrometry
Ler: Landsberg erecta
MS: Murashige and Skoog
MW: molecular weight
qRT-PCR: quantitative reverse transcription PCR
TFA: trifluoroacetic acid.
