# Supplementary material for: A salt-regulated peptide derived from the CAP superfamily protein negatively regulates salt-stress tolerance in Arabidopsis
Source: J Exp Bot. 2015 Jun 20;66(17):5301–13. doi: 10.1093/jxb/erv263 (PMC4526916; doi:10.1093/jxb/erv263)
Supplement: Supplementary Data [file supp_66_17_5301__index.html]

A salt-regulated peptide derived from the CAP superfamily protein negatively regulates salt-stress tolerance in Arabidopsis — A salt-regulated peptide derived from the CAP superfamily protein negatively regulates salt-stress tolerance in Arabidopsis — Supplementary Data 

# A salt-regulated peptide derived from the CAP superfamily protein negatively regulates salt-stress tolerance in *Arabidopsis*

## Supplementary Data

Data files

- Supplementary Data - Supplementary Data
- Supplementary Data - Supplementary Data
- Supplementary Data - Supplementary Data
- Supplementary Data - Supplementary Data
- Supplementary Data - Supplementary Data
