# Supplementary material for: A salt-regulated peptide derived from the CAP superfamily protein negatively regulates salt-stress tolerance in Arabidopsis
Source: J Exp Bot. 2015 Jun 20;66(17):5301–13. doi: 10.1093/jxb/erv263 (PMC4526916; doi:10.1093/jxb/erv263)
Supplement: Supplementary Data [file supp_erv263_jexbot147041_file005.pdf]

SUPPLEMENTAL MATERIALS AND MATHODS

Germination Assay

Seeds were surface sterilized and sown on 1/2 MS medium supplemented with various concentrations of NaCl. After 3 days of stratification, seeds were exposed to a 16-h-light regime at 22°C. The germination of seeds was scored as positive when the tip of the radical fully penetrated the seed coat.

RNA Extraction and RT-qPCR Analysis

Ten seedlings were grown vertically on the mesh attached on 1/2 MS medium for 10 days. Seedlings with the mesh were transferred to a new petri dish and then covered by buffer-saturated filter papers (Whatman; Cat no. 1442-090) with 1/2 MS liquid medium in the presence and absence of 125 mM NaCl. Seedlings sampled at the indicated time points were stored at -80°C until use.

Total RNA was isolated with TRIzol reagent (Invitrogen, Carlsbad, CA, USA) according to the manufacturer's recommendations followed by DNase treatments at 37°C for 15 minutes with RQ1 RNase-free DNase (Promega, Madison, WI, USA) to remove the contamination of genomic DNA. Equal volume of Phenol:Chloroform:Isoamyl Alcohol (25:24:1; Sigma, St. Louis, MO, USA) was added to remove the contaminants of protein and DNA, and total RNA was precipitated by ethanol. Five micrograms of total RNA was used for first-strand complementary DNA synthesis in 20 μL reaction with SuperScript III reverse transcriptase (Invitrogen). For RT-PCR, 1 μL of synthesized cDNA was directly used in a total amount of 20 μL reaction to determine the transcriptional level of *PROAtCAPE1*. For RT-qPCR, 50x dilution of the synthesized cDNA was prepared, and 3 μL of the diluted cDNA was mixed with Fast SYBR

Green Master Mix (Applied Biosystems, CA, USA) in a total amount of 10 μL for subsequent RT-qPCR analysis in ABI 7500 Fast Real-Time PCR system (Applied Biosystems). Gene-specific primers were designed from the 3' untranslated region of Arabidopsis genes by Primer Express 3.0.1 (Applied Biosystems). The transcript of *ACTIN2* was used for normalization.

Western Blot Analysis

Ten-day-old CAPE1ox^{CNYD} seedlings were subjected to medium supplemented with and without 125 mM NaCl for various time points. The collected samples were crushed with YSZ Grinding Media (EE-TEC, EZEAG0500) in 2 mL eppendorf tubes by sonication (KURABO, SH-48). During the preparation, the samples were submerged in liquid nitrogen for rapid freezing. The samples were then homogenized in extraction buffer (50 mM Tris-HCl, pH 7.5, 150 mM NaCl, 20 mM EDTA, 1% [w/v] SDS and 10% [v/v] glycerol) at 95°C for 5 min. After centrifuging at 12,000 rpm for 10 min, the supernatant was transferred to a new eppendorf tube. Fifty micrograms of total proteins were loaded on 10% SDS-polyacrylamide gels and analyzed by western blotting. The recombinant PROAtCAPE1 tagged eYFP was detected by anti-GFP antibody (Roche; 11814460001), and horseradish peroxidase-conjugated anti-mouse antibody (Invitrogen; 616520) was used as a secondary antibody. To confirm the equal loading of total proteins, an anti-α-tubulin antibody (Sigma-Aldrich; T5168) was subsequently used to probe the same blot.

Supplemental Table 1. Effect of selected abiotic stresses on expressions of *PROAtCAPEs*[a].

All data used were generated on Affymetrix GeneChip ATH1 22K platform. –, Reduced expression; +, increased expression after treatment; =, no significant effect on expression (defined as $P \geq 0.05$ and/or fold-change ≤ 1.5 in expression levels compared with control); ND, *PROAtCAPE* gene is unavailable in Genevestigator.

Gene / Stress[b]												*PRB-1*				*PR-1*	
	PROAtCAPE1		*PROAtCAPE2*		*PROAtCAPE3*		*PROAtCAPE4*		*PROAtCAPE5*	*PROAtCAPE6*		*PROAtCAPE7*		*PROAtCAPE8*		*PROAtCAPE9*	
	Root	Shoot	Root	Shoot	Root	Shoot	Root	Shoot		Root	Shoot	Root	Shoot	Root	Shoot	Root	Shoot
Salt	-	+	=	-	-	+	-	=	ND	-	=	=	=	=	=	=	-
Oxidative	=	=	=	=	=	=	=	=	ND	=	-	+	=	=	=	=	-
Osmotic	=	=	=	=	-	=	-	=	ND	-	=	=	=	=	=	=	=
Drought	=	=	=	-	-	=	=	=	ND	-	=	=	=	=	=	=	-
Cold	=	=	=	+	-	=	=	=	ND	=	=	+	=	=	=	=	-

[a]The analysis was done by using Genevestigator[29] microarray database.

[b]Arabidopsis Col-0 were treated with the following stresses and sampled for 0.5, 1, and 3 h:

Wounding: punctured with pins; Salt: 150 mM NaCl; Oxidative: 10 μM Methyl Viologen; Osmotic: 300 mM mannitol; Drought: exposed to the air stream for 15 min and returned to the climate chamber; Cold: 4°C.

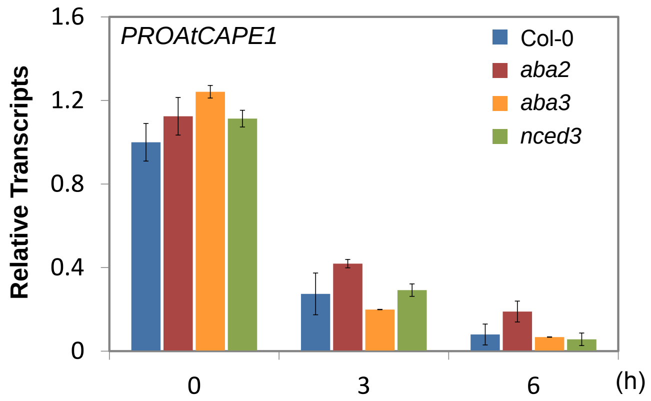

Supplemental Fig. S1. Levels of *PROAtCAPE1* transcripts in the mutants of ABA biosynthesis genes under salinity. Ten-day old wild-type (Col-0) seedlings were subjected to 150 mM NaCl for 0, 3, and 6 h. Relative transcripts indicate the normalized *PROAtCAPE1* level (*PROAtCAPE1*/*ACTIN2*) from each sample is comparing with that in WT upon medium (1/2 MS) alone at 0 h. The bars shown here are the average from two technical repeats.

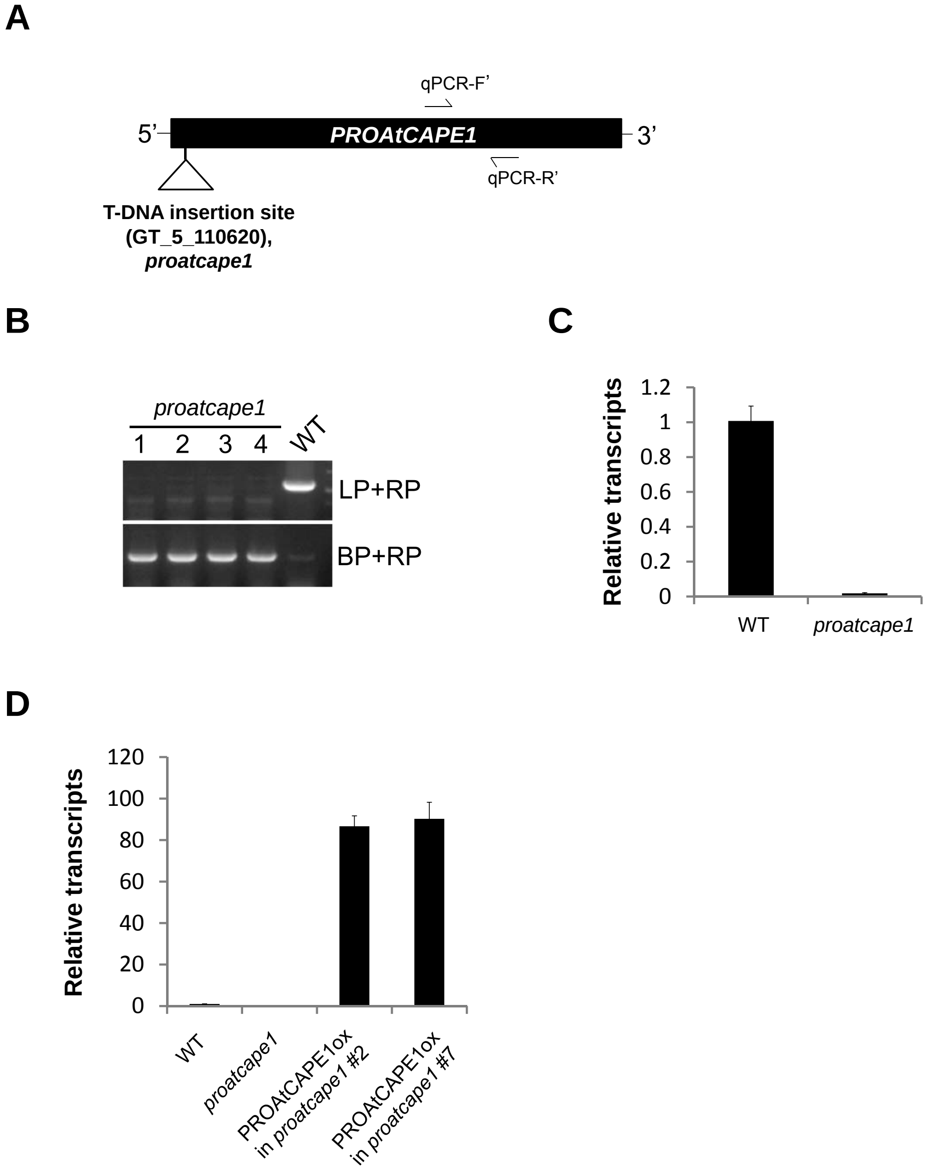

Supplemental Fig. S2. Transcripts of *PROAtCAPE1* in transgenic plants.
(A) Schematic figure represents T-DNA insertion target sites. Filled black boxes: exon; triangle: T-DNA insertion site; arrowheads: primers, qPCR-F’ versus qPCR-R’, used to detect *PROAtCAPE1* transcripts by RT-qPCR. (B) Determination of homozygous *proatcape1* mutants by genotyping. (C) Transcriptional levels of *PROAtCAPE1* in WT (Ler) and *proatcape1* mutant. Relative transcripts indicate the normalized *PROAtCAPE1* level (*PROAtCAPE1*/*ACTIN2*) from each sample is comparing with that in WT. The bars shown here were the average from three biological repeats. Error bars indicated the means ± SE. (D) The relative transcripts of *PROAtCAPE1* in PROATCAPE1ox in *proatcape1* transgenic plants.

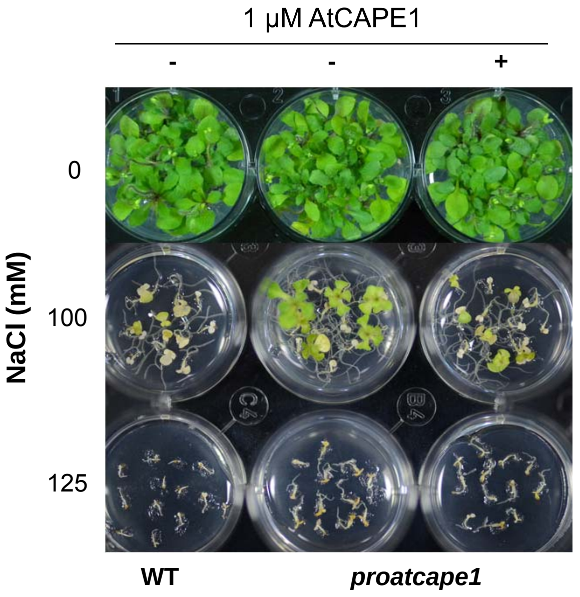

Supplemental Fig. S3. AtCAPE1 negatively regulates salt tolerance response.
Growth and survival of wild type and *proatcape1* mutant seedlings. Seedlings of wild type (Ler) and the *proatcape1* mutants were grown for 20 days in 1/2 MS media supplemented with the indicated concentrations of NaCl in the absence (-) and presence (+) of 1 µM synthetic AtCAPE1 peptide.

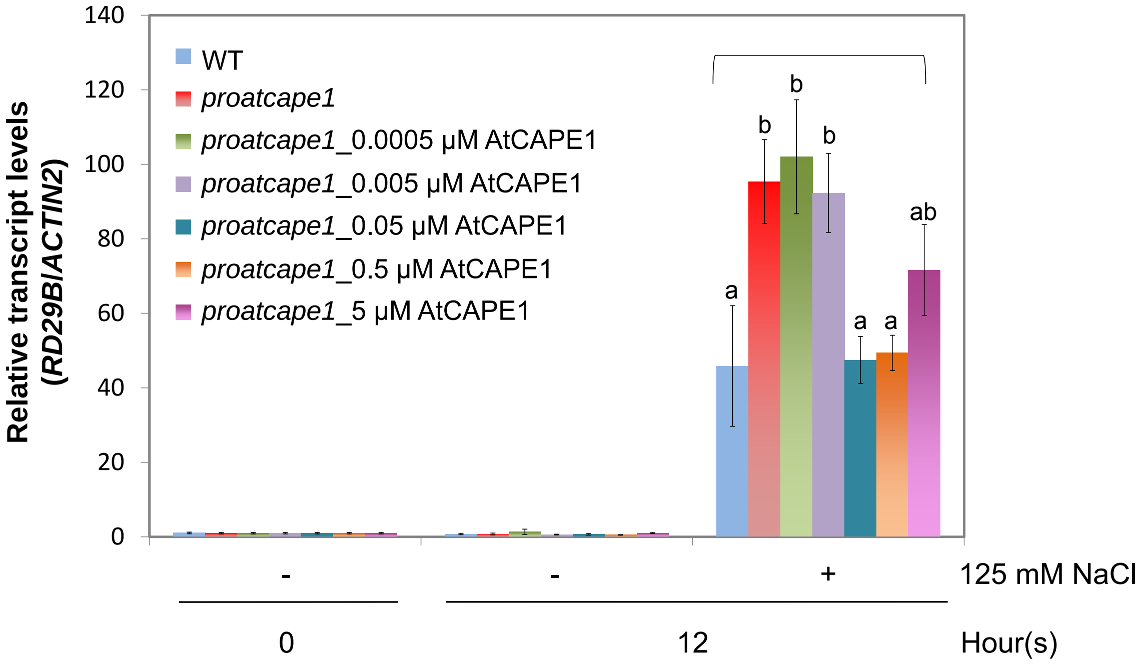

Supplemental Fig. S4. Application of AtCAPE1 peptide reduces the transcript levels of *RD29B* under salinity.
Transcript levels of *RD29B* were determined. Ten-day old wild-type Ler were treated with 125 mM NaCl for 12 hours while *cape1* mutants were treated with 125 mM NaCl supplemented without or with various concentrations of peptides. For the mutants co-treated with salt and peptide, the peptides were pretreated 6 hours before the co-treatments. The relative levels indicate the normalized *RD29B* transcript from each treatment was compared with the expression of *RD29B* in wild type at 0 hour. The bars shown here are the average from four biological repeats. Error bars indicate the means ± SE. The different lowercase letters (a–b) indicate significant differences ($P \leq 0.05$) between treatments.

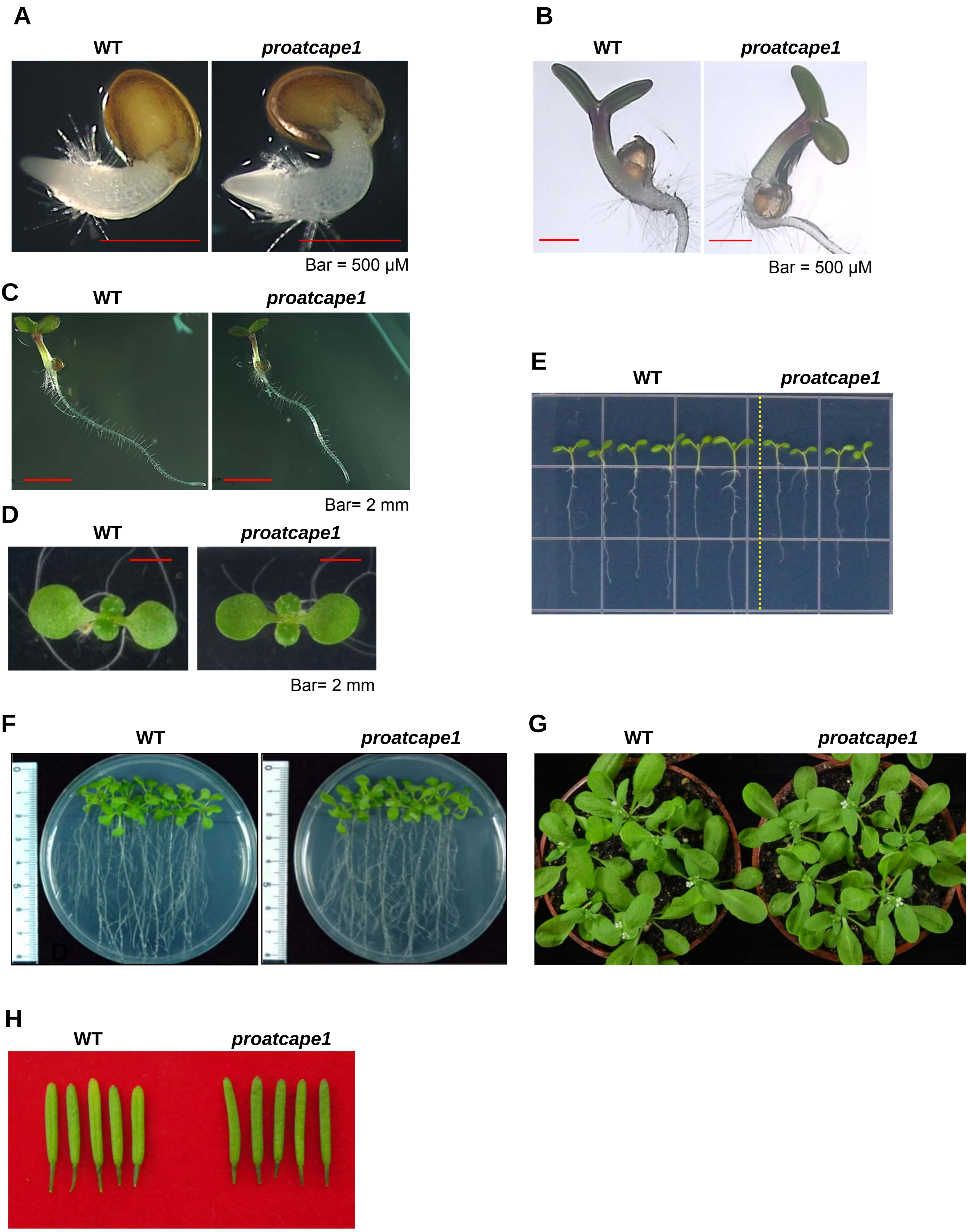

Supplemental Fig. S5. Phenotypic investigation of wild-type (WT) Ler and *proatcape1* mutant.
(A) Germination on day one. (B) Emergence of hypocotyl and cotyledons on day two. (C) Root development on day three. (D) Leaf growth of seedlings on day seven. (E) Primary root of seedlings on day seven. (F) Fifteen-day-old seedlings. (G) Plants at inflorescence stage (around 30-day-old). (H) Siliques from adult plants.

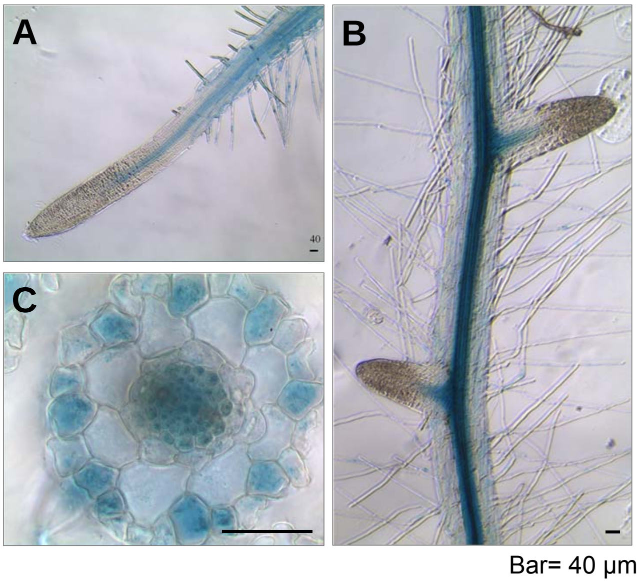

Supplemental Fig. S6. Expressions of *PROAtCAPE1* in roots.

(A) GUS staining showed promoter activities of *PROAtCAPE1* in elongation to maturation zone of primary roots, (B) stele, and tricoblast (C).

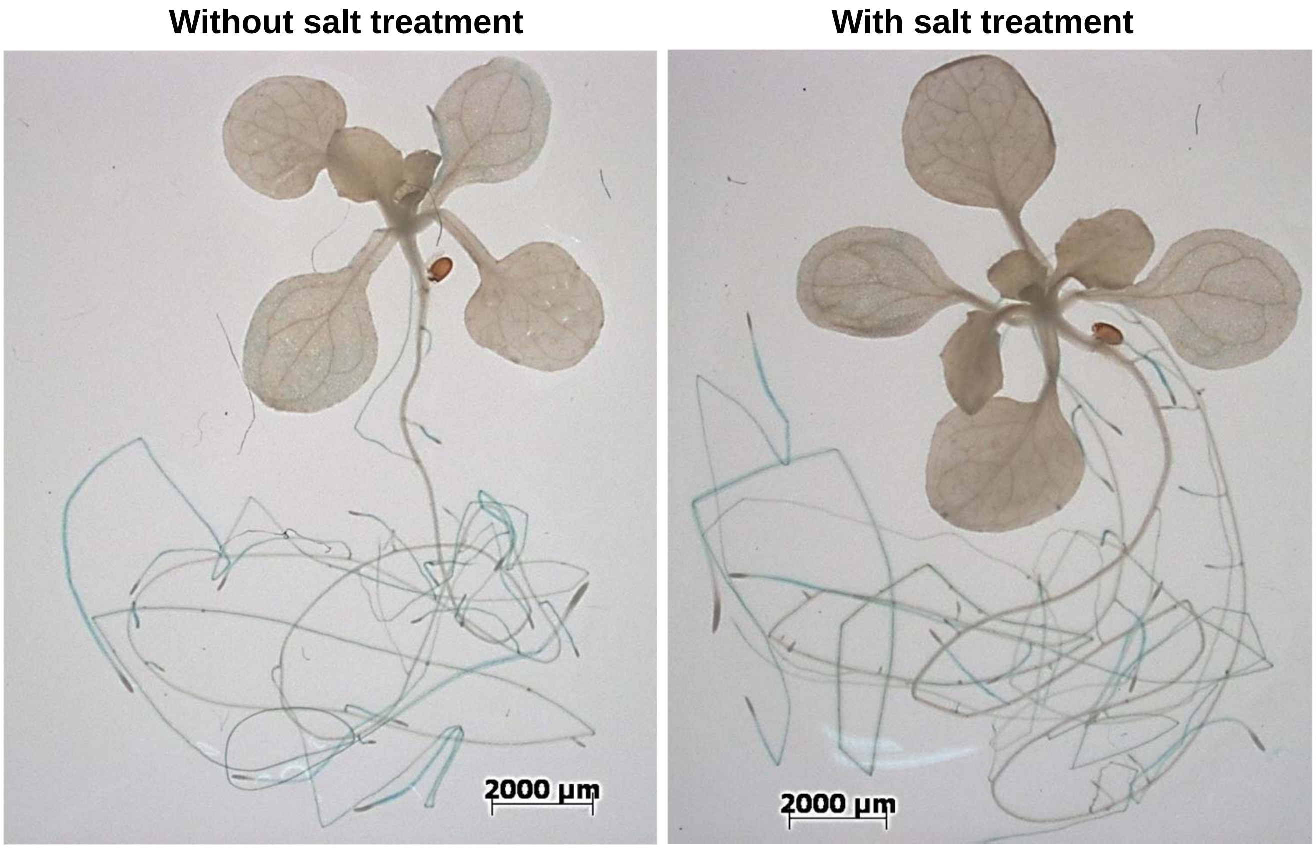

Supplemental Fig. S7. GUS-staining assay revealed the *PROAtCAPE1* promoter activity in the shoots and roots upon salt stress.
p*PROAtCAPE1*:GUS transgenic plants were grown vertically for 10 days. Seedlings with the mesh were transferred to a new petri dish and then covered by buffer-saturated filter papers with 1/2 MS liquid medium in the presence and absence of 125 mM NaCl for 24 h. Red arrowhead indicates the tissue with GUS expression.

Name	purpose	sequence (5'-->3')
AtCAPE1-F'	AtCAPE11 coding region cloning/ gene expressions for RT-PCR	ATGTTTCATAAGGTTGCTATTGAAAC
AtCAPE1-R'		TCAATATGGTCTAGCTCCAATGTAATTG
AtCAPE1_SalI-F'	AtCAPE1 promoter cloning	TTTGGTCGACATATGAAAATCTCCTCTCC
AtCAPE1_SalI-R'		TATGGTTCCATACATTTTAGAAAAGTCGAC
AtCAPE1_LP	Genotyping	AGCTAGGGTTGATTAGGTCCG
AtCAPE1_RP		TTGGTTATGCGATCAAAAAGG
AtCAPE1_qPCR_F'	For AtCAPE1 qPCR	TCAGGCGGTTGCGATGT
AtCAPE1_qPCR_R'		CGGACCATGGCACGAGTT
35S_F'	Confirmation for plants with overexpresed AtCAPE1 tagged eYFP	CCTTCGCAAGACCCTTCCTC
eYFP_R'		AGCTGGTCCTACCCGTGGTG
ABI5_qPCR_F'	Selected salt responsive gene determination	CAGCTGCAGGTTCACATTCTG
ABI5_qPCR_R'		CACCCTCGCCTCCATTGTTAT
ALDH7B4_qPCR_F'		TGGAAACAGTACATGCGTCGAT
ALDH7B4_qPCR_R'		CCTTGCGCTAGAGGTAACTCGTT
AREB1_qPCR_F'		GGATCATGGAAATGCAAAAGAAT
AREB1_qPCR_R'		TTGGACCTCCTTGCAGAAGATT
GolS2_qPCR_F'		GCCACAAGAAGCAACAGACACT
GolS2_qPCR_R'		CGCACCGGCTTCAGACA
P5CS1_qPCR_F'		CTTGTGATACGGATATGGCAAAGCG
P5CS1_qPCR_R'		CCTTGGTCCACCATACAAAGTGACTCC
RD20_qPCR_F'		TCCAGATACAGAGCATCCGAATG
RD20_qPCR_R'		CTCCCAAGGATAGACGATTCCATC
RD22_qPCR_F'		GTGGAGAGGAGAAGTATTGTGCGA
RD22_qPCR_R'		CGGTGCGTTCTTCTTAGCCA
RD29B_qPCR_F'		GTGAAGATGACTATCTCGGTGG
RD29B_qPCR_R'		CACCACTGAGATAATCCGATCC
ACTIN2_qPCR_F'	Internal control for qPCR	GGTAACATTGTGCTCAGTGGTGG
ACTIN2_qPCR_R'		GGTGCAACGACCTTAATCTTCAT

Supplemental Table 2. Primers used in this study.
